# Five New Species of the Lichen-Forming Fungal Genus *Peltula* from China

**DOI:** 10.3390/jof8020134

**Published:** 2022-01-28

**Authors:** Qiuxia Yang, Xiangmin Cheng, Tingting Zhang, Xinzhan Liu, Xinli Wei

**Affiliations:** State Key Laboratory of Mycology, Institute of Microbiology, Chinese Academy of Sciences, Beijing 100101, China; yangqx@im.ac.cn (Q.Y.); 18435122279@163.com (X.C.); tingtingzhang813@163.com (T.Z.); liuxinzhan@im.ac.cn (X.L.)

**Keywords:** Lichinales, molecular systematics, new to science, Peltulaceae, taxonomy

## Abstract

The genus *Peltula* is an important cyanobacterial lichen group. We performed a taxonomic study on the *Peltula* from China using phylogenetic analysis based on three gene loci (ITS, nuSSU, nuLSU) together with additional species delimitation analyses by ABGD, bPTP and GMYC approaches and the phenotypic characteristics. Five new species (*Peltula lobulata*, *P. polycarpa*, *P. polyphylla*, *P. pseudoboletiformis* and *P. submarginata*) were found and described. *Peltula lobulata* is diagnostic in its small thallus with plenty of lobules, rolled down and irregularly lobed margins, and uneven cracked surfaces. *Peltula polycarpa* has convex and rolled down lobes and numerous apothecia with a thalloid rim covering the whole lobe, and it can be distinguished from fertile *P. farinosa* (southern Switzerland) by a bright olive-green and epruinose surface, and the absence of isidia. *Peltula polyphylla* is differentiated from any other known *Peltula* species by a very small polyphyllous thallus composed of abundant olive-brown to olive-black small lobes growing tightly and sometimes anastomosing and attaching to the substrate by a large and strong umbilical cluster. *Peltula submarginata* is similar to *P. marginata* but differs in the presence of encircled epinecral and algae layers, and the absence of a lower cortex. *Peltula pseudoboletiformis* is different from the similar species *P. boletiformis* in greener lobes, more yellow–green umbilici and certain phylogenetic differences. Moreover, a key to the species of *Peltula* in China is also provided here.

## 1. Introduction

The lichen family Peltulaceae, affiliated to Lichinales, Lichinomycetes, Ascomycota, has been reported to have more than 50 species all over the world thus far and 14 known in China [1]. *Peltula* Nyl. is the only genus in this family and is symbiosed with the cyanobacterial photobiont known as *Chroococcidiopsis*, *Myxosarcina*, *Gloeocapsa* [2,3,4,5] or Chroococcales [6,7,8]. Through isolation and culture of lichen specimens and the construction of phylogenetic trees, Jung et al. [9] identified cyanobionts of *Peltula* as *Aliterella* and *Compactococcus* gen. nov., *Pseudocyanosarcina* gen. nov., and an unknown species, but did not find *Chroococcidiopsis,* which is the only known unicellular terrestrial genus and could be considered robust support to delimit the genus *Peltula*.

The genus *Peltula* is comprised of species with an olive-green peltate, squamulose to subfruticose thalli attached to the substratum by umbilicus or rhizines, immersed apothecia, a gelatinous sheath on the ascus and numerous spores per ascus [10,11]. It is currently subdivided into six growth forms of thallus, viz. peltate-umbilicate, squamulose-semifruticose, squamulose-compound, subfoliose-compound, crustose-areolate and crustose based on the revision of Peltulaceae [12]. The most diagnostic characters within *Peltula* refer to anatomical structures. The upper cortex is generally absent in *Peltula* species, with the epinecral layer existing instead, except for *P. impressula*, which has a translucid upper cortex, as well as *P. farinosa* [5] and *P. sonorensis* [4,13] with a primitive upper cortex; the medulla is often composed of interwoven or cellular hyphae with a large air space existing in the subfruticose thallus; and the lower cortex in most species clearly consists of 3–8 layers of large cells. Most of the species in *Peltula* have immersed apothecia, while *P. auriculata* and *P.* *lobata* are characterized by adnate type and *P. imbricata* with a sessile type [6,7,14]. In chemistry, only two closely related yellow pigments, myeloconone D_1_ and D_2_, have been detected from *Peltula langei* Büdel ex Elix [15].

Previous reports of *Peltula* in China mainly focused on Hong Kong [16,17], Taiwan [18], Gansu [19,20] and Inner Mongolia [21]. This study is based on an additional survey of *Peltula* on the national scale, including both the typical arid and semi-arid areas such as Inner Mongolia, Gansu, Ningxia and Qinghai, and some humid and semi-humid areas such as Anhui, Beijing, Hebei, etc. Surprisingly, Beijing is found to be abundant in *Peltula* species diversity for the first time, and all the five new species with umbilici, rhizines or stalks attaching to substrates, such as soil or rock surfaces, are described from here.

## 2. Materials and Methods

### 2.1. Taxon Sampling

**Figure 1:**
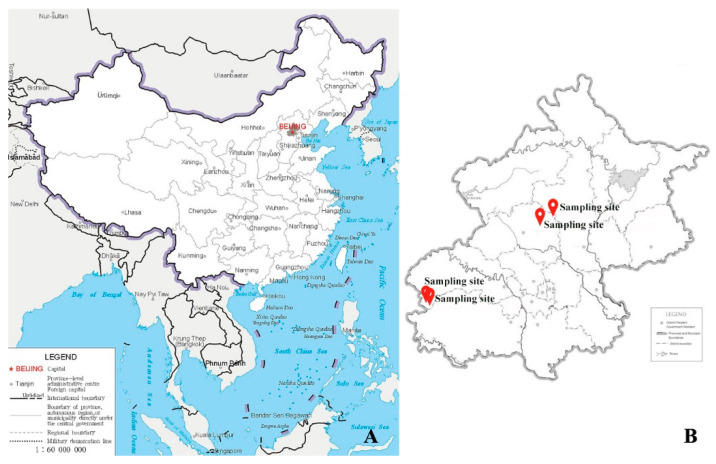
Distribution of the five new *Peltula* species in China. (**A**). The collection area in China, shown in grey colour and marked in red font. (**B**). The four sampling sites in the collection area.

Fifteen specimens for this study were collected from Beijing, capital of China (see Figure 1). The voucher specimens were deposited in the lichen section of fungarium of Institute of Microbiology, Chinese Academy of Sciences (HMAS-L). A LEICA M125 and LEICA DFC450 dissecting microscopes were used for the morphological studies. The internal morphology of the lichen thallus and ascomata was studied using free-hand sections. A Zeiss Axioscope2 compound microscope with a Zeiss Axio Imager A2 was used for the anatomical studies, and a Zeiss AxioCam MRc5 camera was used for taking photographs. Spot tests were performed using K (10% aqueous solution of potassium hydroxide) and IKI (1% aqueous iodine solution with 10% aqueous potassium hydroxide). Lichen substances were examined using standardized thin layer chromatography (TLC, solvent C) [22].

### 2.2. DNA Extraction, PCR and Sequencing

Fourteen fresh specimens were chosen for DNA extraction in this study. The extraction procedure followed a modified CTAB method [23]. PCR was performed to amplify three gene loci: nuclear ribosomal DNA internal transcribed spacer (ITS), small subunit (nuSSU) and large subunit (nuLSU). The PCR primers and procedures of nuSSU and nuLSU followed Kauff et al. [12]. ITS was amplified using the primers ITS4 and ITS5 [24] in 25 µL reactions containing 12.5 µL 2 × Taq PCR MasterMix® (Beijing Jiangchen Biotechnology Co., Ltd., Beijing, China), 1 µL each primer solution (10 µM), 9 µL ddH_2_O and 1.5 µL genomic DNA, and the PCR cycling conditions comprised of an initial denaturation at 94 °C for 2 min; 33 denaturation cycles at 94 °C for 30 s, annealing at 55 °C for 30 s, extension at 72 °C for 1 min 10 s; and a final extension at 72 °C for 2 min. The target PCR products were checked by electrophoresis on 1% agarose gels and then sequenced in Ruibio BioTech Co., Ltd. (Beijing, China) and BioSune (Shanghai) (Beijing, China). The new sequences generated for this study were deposited in GenBank. A total of 162 DNA sequences including 37 new sequences (14 ITS, 14 nuLSU and 9 nuSSU) generated for this study under accession numbers MT499282–MT499304 and MT499313–MT499326 were used in this study (Table 1). *Lichinella iodopulchra* (Lichinales) was chosen as the outgroup. All sequences of three loci (ITS, nuSSU, nuLSU) were aligned separately using ClustalW Multiple Alignment [25] in BioEdit v7.0.5 [26]. The program Gblocks v0.91b [27,28] was used to remove regions of alignment uncertainty, using options for a “less stringent” selection on the Gblocks web server (http://molevol.cmima.csic.es/castresana/Gblocks_server.html, accessed on 1 May 2021).

### 2.3. Congruence among Loci

To test the level of congruence among loci, highly supported clades (equal to or more than 75% bootstrap) from single-gene trees were compared and assessed [29,30]. When there was no conflict using a 75% bootstrap value threshold, in situations where a monophyletic group was supported with bootstrap values ≥75% at one locus and the same group of taxa was supported (≤75%) as nonmonophyletic with another locus, then the group was assumed to be congruent, and the data set was concatenated [30]. Each locus was subjected to a randomized accelerated maximum likelihood (RAxML) analysis, involving 1000 pseudoreplicates with RAxML-HPC BlackBox 8.2.6 [31] on the Cipres Science Gateway (http://www.phylo.org, accessed on 1 May 2021), and all three single-locus RAxML trees were compared. We removed the conflicting gene sequences based on the significant topological differences and repeated the test until no further conflicts could be detected. The best model for the three single genes used in the phylogenetic analysis was identified using PartitionFinder 2 [32]. The results were visualized with FigTree 1.4.2.

### 2.4. Phylogeny of the Genus Peltula

Phylogenetic analyses of *Peltula* were performed using the concatenated data set using RAxML-HPC v. 8.2.6 [31] and MrBayes v.3.2.6 [33,34] on the Cipres Science Gateway (http://www.phylo.org, accessed on 1 May 2021). The GTR + I + G model was selected in both ML and Bayesian analyses with 1000 pseudoreplicates. Two parallel Markov chain Monte Carlo (MCMC) runs were performed in MrBayes, each using 8 million generations and sampling every 1000 steps. A 50% majority-rule consensus tree was generated from the combined sampled trees of both runs after discarding the first 25% as burn-in. Tree files were visualized with FigTree v.1.4.2 (http://tree.bio.ed.ac.uk/software/figtree/, accessed on 1 May 2021). The multilocus data alignment file was submitted to TreeBASE (submission ID 26385).

### 2.5. Species Delimitation Analyses

Three species delimitation methods, Automatic Barcode Gap Discovery (ABGD) [35], a Bayesian implementation of the Poisson tree process model (bPTP) [36] and the General Mixed Yule Coalescent (GMYC) approach [37,38], were used to circumscribe species boundaries in the genus *Peltula* from the ITS and nuLSU sequence alignments.

ABGD analysis was performed using the ABGD webserver (http://wwwabi.snv.jussieu.fr/public/abgd/abgdweb.html, accessed on 20 October 2021). We used default parameters except for using a Pmax at 0.01 and a relative gap width of 1.5 with the Jukes-Cantor model (JC69). The PTP model is intended for delimiting species in three gene-locus (ITS + nuSSU + nuLSU) molecular phylogenies and provides an objective approach for delimiting putative species boundaries that are consistent with the phylogenetic species criteria. We used the bPTP web server (http://species.h-its.org, accessed on 20 October 2021) [36] to delimit putative species groups using the concatenated topology as the input tree and implementing default settings. The GMYC method aims to detect shifts in branching rates between intra- and inter-specific relationships. Within a likelihood framework, it uses chronograms to compare a null model under which the whole sample belongs to a single species and hence follows a coalescent process and an alternative general mixed Yule coalescent (GMYC) model. The latter combines equations describing branching patterns within and among lineages. A likelihood ratio test (LRT) was used to evaluate whether the null model can be significantly rejected. If the GMYC model fits the data significantly better than the null model, the threshold T allows for the estimation of the number of species present in the data set. First, ultrametric trees were estimated using the program BEAST v2.6.3 [39]. We ran two independent Markov Chain Monte Carlo (MCMC) chains for 15 million generations under the coalescent model with a constant population size and a constant clock as the tree prior. Default values were used for the remaining priors. The outputs were diagnosed for convergence using TRACER v.1.7.2 [40] after removing 10% of the samples as a burn-in. The effective sample size (ESS) values greater than 200 were considered a good indicator. Tree files from two independent runs were combined using LogCombiner [39]. A consensus tree was generated using TreeAnnotator 1.8.2 [39] after discarding the first 3000 trees. The GMYC analysis was performed on an ultrametric consensus tree under the single-threshold model using the SPLITS package [38] available for R 4.0.5 [41].

## 3. Results

### 3.1. Phylogenetic Analysis

**Figure 2:**
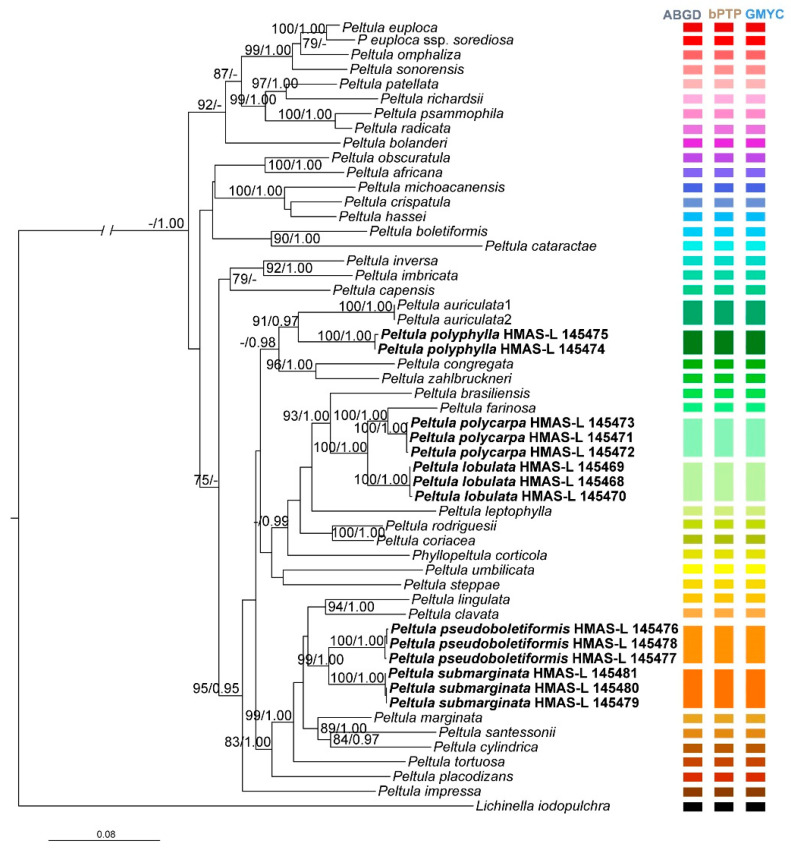
The RAxML tree of *Peltula* species based on the concatenated ITS + nuSSU + nuLSU data set. The numbers in each node represent bootstrap support (BS) and posterior probability (PP) values. BS values ≥ 75 and PP values ≥ 0.95 were plotted on the branches of the tree. The clades corresponding to the new species are in bold, which indicates that these sequences were newly generated for this study. Scale in 0.08 substitution per site. Three species delimitation analysis results are listed on the right side of the tree, among which different colour patches correspond to the different species recognized by the software, and the same colour patches refer to the same species.

The aligned matrix contained 3380 unambiguous nucleotide position characteristics for the full data set of 54 members. BI and ML phylogenetic trees of *Peltula* were constructed, and they had similar topological structures. The RAxML tree is shown in Figure 2 with both bootstrap support (BS) and posterior probability (PP) values of BI analysis. In the tree, all the *Peltula* species clustered into a monophyletic clade and obviously separated from the outgroup (PP = 1), within which five well-supported (BS = 100, PP = 1.00) branches corresponding to the five new species were included. The multilocus phylogenetic analysis highly supports (BS100/PP1.00) that *Peltula farinosa*, *P. polycarpa* and *P. lobulate* clustered closely but also distinctly (see Figure 2), and they can be easily distinguished in morphology. The BI phylogenetic tree and three single-gene-locus RAxML trees are shown in Figures S1–S4.

The results of species delimitation analyses are shown in Figure 2. Different colour patches correspond to different species, and the same colour patches refer to the same species. The ABGD analysis, based on the concatenated data set, provided evidence supporting the species delimitation scenario similar to the phylogenetic results, for example, *Peltula farinosa*, *P. polycarpa* and *P. lobulata* are delimited into three separate species, but the same species *Peltula euploca* and *P. eupcola* ssp. *sorediosa* are delimited into two different ones (Table S1). The tree-based bPTP analysis obtained the same results as ABGD analysis (Table S2), and the GMYC analysis provided further and stronger support (most of the posterior probability = 1.0) for delimiting all the species (Table S3).

### 3.2. Taxonomy

The genus *Peltula* is a cyanobacterial lichen group. It should be recognized that the cyanobiont morphology described here refers to the lichenized state in thallus not the free-living form in nature [42]. A key to the species of *Peltula* is listed in Table 2.

**Figure 3:**
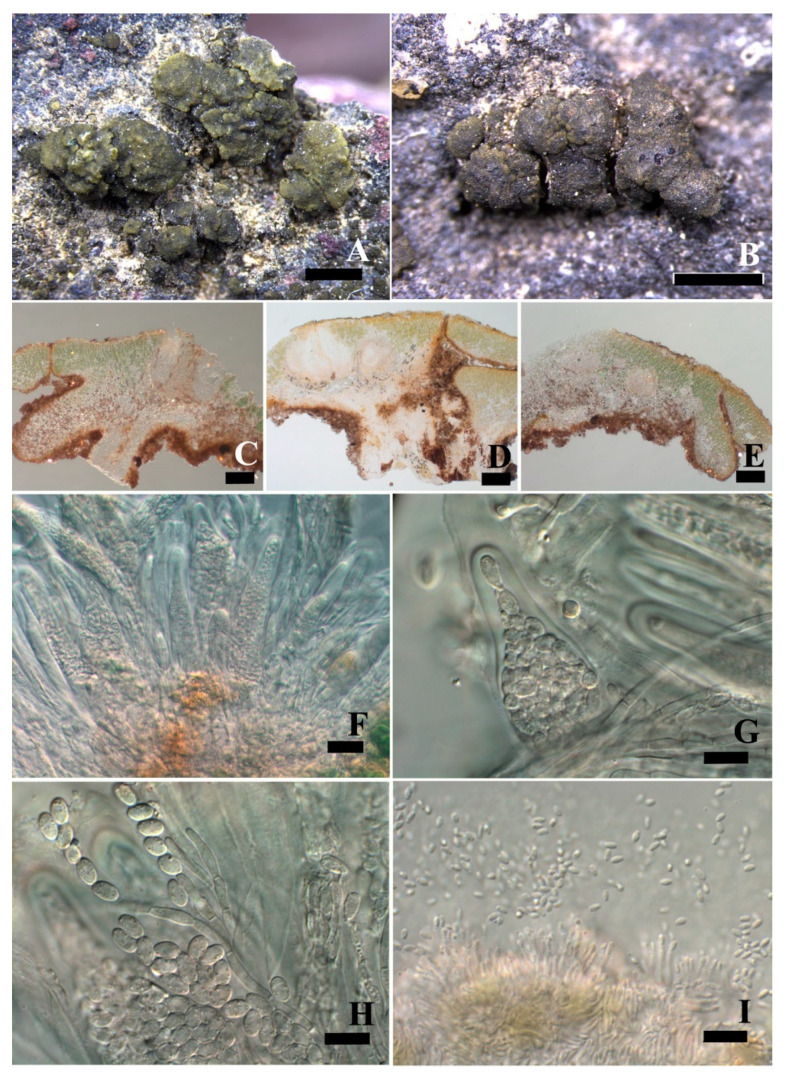
The thallus habit and the anatomic structure of *Peltula lobulata* (holotype). (**A**,**B**). Squamulose thallus and immersed apothecia. (**C**–**E**). Thallus section. (**F**). Polysporous ascus. (**G**). Asci with lacerate gelatinous sheeth. (**H**). Elliptical ascospores. (**I**). Conidia. Bars: (**A**,**B**) = 1 mm, (**C**–**E**) = 100 μm, (**F**) = 20 μm, (**G**–**I**) = 10 μm.

***Peltula lobulata*** Q.X. Yang & X.L. Wei, **sp. nov.** (Figure 3).

**MycoBank:** MB 840880.

**Etymology:** The epithet ‘*lobulata*’ refers to the distinctive character of plenty of lobules in this species.

**Typus:** CHINA, Beijing, Changping District, Mangshan National Forest Park, 40°15′ N, 116°17′ E, alt 450 m, on rocks, 31 May 2019, W.C. Wang & Q.X. Yang 20,191,471 (HMAS–L145468, holotype).

**Diagnosis:** It is characterized by many lobules, irregularly lobed margins, and uneven surfaces.

**Description:** Thallus saxicolous, squamulose, up to 2.5 mm in diameter, irregularly rounded, convex to flat, plenty of lobules present at mature thallus; margins rolled down and lobed, paler than thallus; upper surface is dark olive-green, colour uneven, rugose, sometimes cracked, epruinose; lower surface matt, almost black, tightly appressed to the substrate by central short umbilicus; isidia and soredia absent. Thallus 185–300 μm thick, lobules up to 125 μm thick; upper cortex not developed but with a yellowish epinecral layer, 5 μm thick; photobiont layer 56–105 μm thick, cyanobiont unicellular, cells 9.3 × 13.4 μm in diam. in clusters of 2–4 cells; medulla composed of loosely interwoven hyphae and round cells, 35–80 μm thick, hyphae 2 μm wide; lower cortex paraplectenchymatous, 30 μm thick, made up of 3–5 layers of big cells, cells 8 × 5.5 μm in size. Apothecia rare, average 1–2 per squamule, very rarely up to 5, punctiform and immersed, later up to 0.2 × 0.27 mm, thalloid rim absent. Epihymenium yellow brown; hymenium approximately 150 μm tall, paraphyses tips sometimes branched, 1.6–2.3 μm wide, I+ wine red, K−; subhymenium approximately 60–87 μm thick, IKI+ blue after K pretreatment; asci clavate to obclavate, with a lacerate gelatinous sheath, ascus 37–70 × 12–19 μm, more than 60 spores, ascus wall IKI+ blue after K pretreatment; ascospores hyaline, ellipsoid, simple, (4.7) 5.5–8.3 × 2.7–3.8 μm. Pycnidia immersed, cerebriform, ellipsoid, hyaline, 2.1–2.8 × 1.5–1.7 μm.

**Chemistry:** No substances detected by TLC.

**Habitat and distribution:** The species sample was found on sun-exposed red shale and gray sandstone along the road on the low altitude mountain. It is dispersed and not associated with other species and known only in China up to now.

Additional specimens examined: CHINA, Beijing, Changping District, Mangshan National Forest Park, 40°15′ N, 116°17′ E, alt 450 m, on rocks, 31 May 2019, W.C. Wang & Q.X. Yang 20,191,474 (HMAS-L145469); Beijing Dayangshan National Forest Park, 40°18′ N, 116°25′ E, alt 390 m, on rocks, 18 June 2019, Q.X. Yang & W.C. Wang 20,191,639 (HMAS-L145470).

**Notes:** This new species is easily overlooked in the field because the thallus is very small and the colour is very similar to the surrounding environment. It is rather difficult to be identified only based on the external morphology but needs additional anatomical structures and DNA sequences. The thallus of this species is irregular (Figure 3A,B), with plenty of lobules on each squamule as the most diagnostic feature, which is more obvious in section (Figure 3C–F) and rare to be observed previously in *Peltula*. 

**Figure 4:**
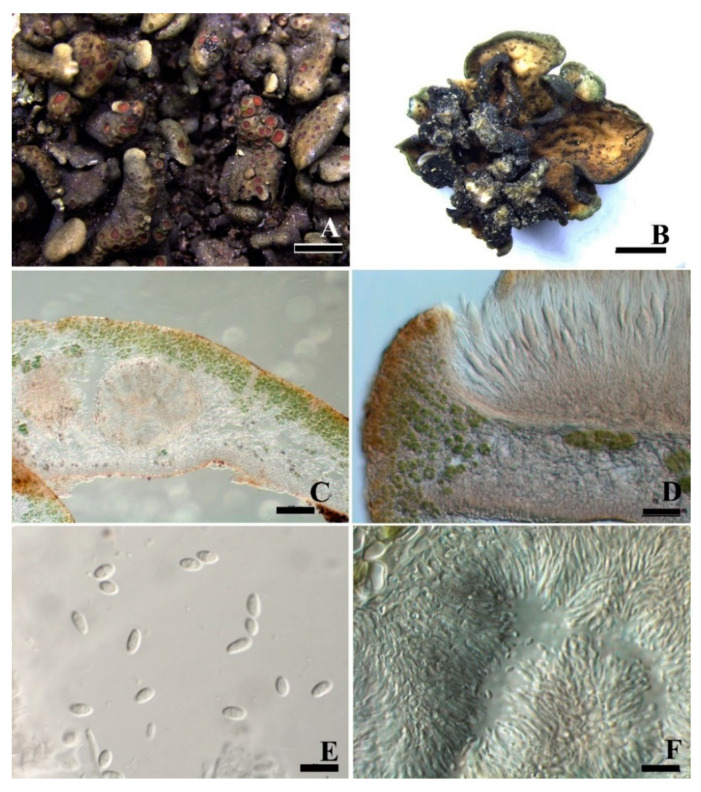
The thallus habit and the anatomic structure of *Peltula* *polycarpa* (holotype). (**A**). Squamulose thallus and apothecia with thalloid rim. (**B**). Concave lower surface of thallus. (**C**). Thallus section with endolichenic fruiting bodies. (**D**). Section of apothecium. (**E**). Elliptical ascospores. (**F**). Conidia. Bars: (**A**) = 1.5 mm, (**B**) = 1 mm, (**C**) = 100 μm, (**D**) = 50 μm, (**E**,**F**) = 10 μm.

***Peltula polycarpa*** Q.X. Yang & X.L. Wei, **sp. nov.** (Figure 4).

**MycoBank:** MB 840881.

**Etymology:** The epithet ‘*polycarpa*’ refers to the character of numerous apothecia in this species.

**Typus:** CHINA, Beijing, Mentougou District, Qingshui Town, Baihuashan National Nature Reserve, 39°50′17′′ N, 115°34′24′′ E, alt ca. 1120 m, on rocks, 31 August 2019, X.L. Wei et al. 20,191,855 (HMAS-L145471, holotype).

**Diagnosis:** It is characterized by numerous apothecia with thalloid rims and large discs (to 0.4 mm) and strongly curved thalli.

**Description:** Thallus saxicolous, scattered to confluent, squamulose or squamulose-compound, up to 5 mm in diameter or up to 6.8 mm long and 4.7 mm wide, initially monophyllous of pale yellow tongue-shaped, attached the substrate by the lateral umbilicus, very young squamules nearly adnate, sometimes later polyphyllous by several lobes growing from the same umbilicus or different umbilici aggregated together (Figure 4B); mature lobes convex to bending down, margins invisible due to the deep curvature, unless viewed on the ventral side, which are entire or slightly lobed; upper surface bright olive-green with olive-brown base, epruinose, lobules occasionally superficial; lower surface smooth, bright yellow to yellowish brown; isidia and soredia absent. Thallus 225–300 μm thick; upper cortex not developed but with a yellowish epinecral layer, 4.5–5.5 μm thick; algal layer 70–100 μm thick, algae in clumps of 2 or 4 cells, 11.5–12.8 × 8.3–10.8 μm in diameter; medulla 125–150 μm thick, composed of loosely interwoven hyphae with small air spaces, hyphae 2–2.5 μm wide; lower cortex paraplectenchymatous, 45–55 μm thick, made up of 5–6 layers of big cells, cells 7.5–10 × 5–8.2 μm in size. Apothecia numerous, covered the whole upper surface, completely immersed, punctiform when young, up to 0.4 mm in diam. or 0.35 mm wide and 0.48 mm long with thalloid rim when mature, margin pale yellow or concolorous to the thallus. Epihymenium yellow–brown; hymenium 140–185 μm tall, I+ wine red, K−, paraphyses septate, apices moniliform, anastomosing, 1.9–2.4 μm wide, sometimes expanded to 5–6 μm; subhymenium approximately 60–70 μm thick with a poorly developed excipulum, IKI+ blue after K pretreatment; asci clavate to obclavate, with a lacerate gelatinous sheath, 96–98 × 13–16 μm, more than 100-spored, ascus wall IKI+ blue after K pretreatment; ascospores hyaline, ellipsoid, simple, 5.8–6.6 × 2.9–3.8 μm. Pycnidia immersed, cerebriform; conidia ellipsoid, hyaline, 2.1–2.3 × 1.2 μm.

**Chemistry:** No substances detected by TLC.

**Habitat and distribution:** This species sample was found on granite, which was periodically wetted by water streaks, along the roadside on the mountain. *Peltula polycarpa* co-occurs with weeds, moss, and other lichens, such as *Phaeophyscia* sp. and other species in Lichinales. It is known only in China up to now.

Additional specimens examined: CHINA, Beijing, Mentougou District, Qingshui Town, Baihuashan National Nature Reserve, 39°50′17′′ N, 115°34′24′′ E, alt ca. 1120 m, on rocks, 31 August 2019, X.L. Wei et al. 20,191,856 (HMAS-L145472), 20,191,857 (HMAS-L145473), 20,191,948 (HMAS-L151066).

**Notes:** This new species is very distinctive and can be readily identifiable in the field by its numerous and bright red apothecia with thalloid rims, especially at maturity, its strongly revolute lobe tips and the light colour of its young lobes. The lobes of most species in *Peltula* have been known to be attached the substrate by rhizines or central umbilici; however, the umbilicus of this species is in the lateral position, which is more prominent in a polyphyllous thallus. Phylogenetic analyses confirm that this species is closely related to *P*. *farinosa*. Indeed, this new species looks very similar to fertile *P. farinosa* (southern Switzerland) because they both have large and broad squamules and numerous apothecia with thalloid rims when mature. However, these two species can still be distinguished quickly in morphology. Fertile *Peltula farinosa* has simple gray squamules without lobules, with a pruinose upper surface and sorediate undulated margins [5,43], while *P. polycarpa* is initially pale-yellow and formed by tongue-shaped squamules, later turns into bright olive-green and polyphyllous thalli, characterized by several lobes growing around a same umbilicus or different umbilici aggregating together with strongly convex thalli, occasionally superficial lobules, and an absence of isidia, soredia and pruina. Although the systematic positions of the two species are close, the morphological characteristics and species delimitation analyses support that they are two independent species.

**Figure 5:**
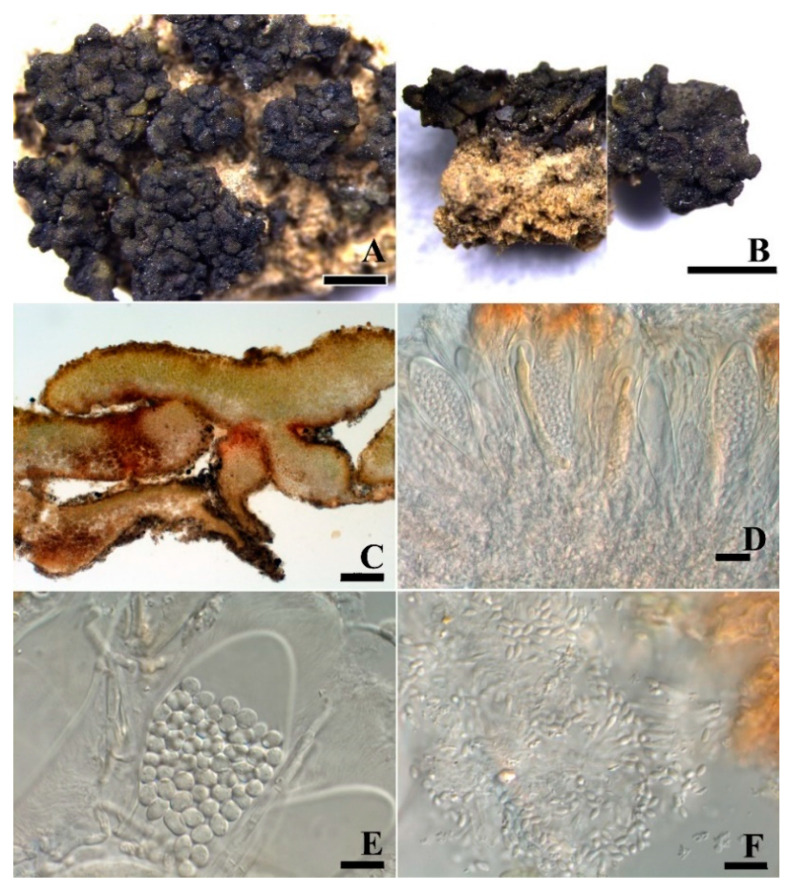
The thallus habit and the anatomic structure of *Peltula* *polyphylla* (holotype). (**A**). Squamulose–compound thallus. (**B**). Side view of thallus and apothecia with thalloid rim. (**C**). Thallus section. (**D**). Polysporous ascus. ©. Elliptical ascospores in an ascus with a lacerate gelatinous sheath. (**F**). Conidia. Bars: (**A**,**B**) = 1 mm, (**C**) = 100 μm, (**D**) = 20 μm, (**E**,**F**) = 10 μm.

***Peltula polyphylla*** Q.X. Yang & X.L. Wei, **sp. nov.** (Figure 5).

**MycoBank:** MB 840882.

**Etymology:** The epithet ‘*polyphylla*’ refers to a polyphyllous thallus of this new species.

**Typus:** CHINA, Beijing, Mentougou District, Qingshui Town, Bamuyan village, behind Xishanhong Inn, 39°51′ N, 115°33′ E, alt ca. 740 m, on soil of rock surface, 1 September 2019, X.L. Wei et al. 20,192,014 (HMAS-L145474, holotype).

**Diagnosis:** The new species is characterized by a polyphyllous thallus composed of abundant olive-brown to olive-black small lobes.

**Description:** Thallus terricolous, polyphyllous, up to 2.5 mm in diameter, lobes up to 0.9 mm in diameter, concave, margins slightly to deeply lobed, undulate and darker; upper surface olive-brown to olive-black, epruinose; lower surface smooth, pale yellowish brown, attached to the substrate by a large and strong umbilicus cluster composed of umbilicus of each lobe; isidia and soredia absent. Thallus 550 μm thick, lobes 130–211 μm thick, upper cortex not developed but with a yellowish epinecral layer, 5 μm thick; algal layer 98.6–104.5 μm thick, algal cells spread to almost the whole medulla, algae in clumps of 1–4 cells, cells 7.4–7.6 × 8.6–12.4 μm in size; medulla thin and unclear, composed of loosely interwoven hyphae and round cells, hyphae 2.8–4.2 μm wide; lower cortex paraplectenchymatous, 26.9–34.6 μm thick, made up of 4–6 layers of big cells, cells 5.8 × 7.3 μm. Apothecia rare, solitary on each lobe (Figure 5B), up to 0.55 mm in diameter, margin concolorous with the thallus. Epihymenium is yellow–brown; hymenium 95–120 μm tall, I+ wine red, K−; subhymenium 85 μm, IKI+ blue after KOH pretreatment; asci clavate to obclavate, 32 × 10–12 μm, with a lacerate gelatinous sheeth, c. 60 spores per ascus, ascus wall IKI+ blue after KOH pretreatment; ascospores hyaline, globose, simple, 3.8–5.7μm. Pycnidia immersed; conidia ellipsoid, hyaline, 3.0–3.3 × 1.5–1.6 μm.

**Chemistry:** No substances detected by TLC.

**Habitat and distribution:** This species sample was found on sun-exposed rocks covered with a thin layer of soil. *Peltula euploca* and *P. placodizans* are on the bare rocks nearby. It is known only in China up to now.

Additional specimens examined: CHINA, Beijing, Changping District, Beijing Dayangshan National Forest Park, 40°18′ N, 116°25′ E, alt 390 m, on rocks, 18 June 2019, Q.X. Yang & W.C. Wang 20,191,645 (HMAS-L 145475).

**Notes:** The thallus of this new species is very small and grows together with other species of *Peltula*, such as *P. euploca* and *P. placodizans*, so it is not easy to be noticed in the field. However, when being observed under a dissecting microscope, this species is readily distinguishable due to its polyphyllous thallus composed of many small lobes. The small lobes grow tightly, sometimes connecting with each other and anastomosing (Figure 5C). The thallus attaches to the substrate by a large and strong umbilical cluster (Figure 5B).

*Peltula polyphylla* is related to *P. auriculata*, but they can be easily distinguished. Thallus of *P. auriculata* attached to the substrate by umbilicus and its lobes have ear-shaped appendices while *P*. *polyphylla* attached to the substrate by a large and strong umbilical cluster with peltate lobes [6]. Phylogenetic analysis indicated that *P*. *polyphylla* and *P. auriculata* clustered into the same clade, which supported that they are closely related but different. The additional species delimitation analyses also defined *P*. *polyphylla* and *P. auriculata* into separate species. Besides, this new species is similar to *P. imbricata* due to having polyphyllous thalli, but the thallus of *P. imbricata* is made up of small imbricate lobes, sometimes with pruinose upper surface, tightly appressed to the substrate, forming small colonies or a continuous crust to 10 mm in diam. While *P. polyphylla* is consists of many peltate and epruinose lobes, attached to the substrate by a large and strong umbilical cluster, and the colonies up to 2.5 mm in diameter [14].

**Figure 6:**
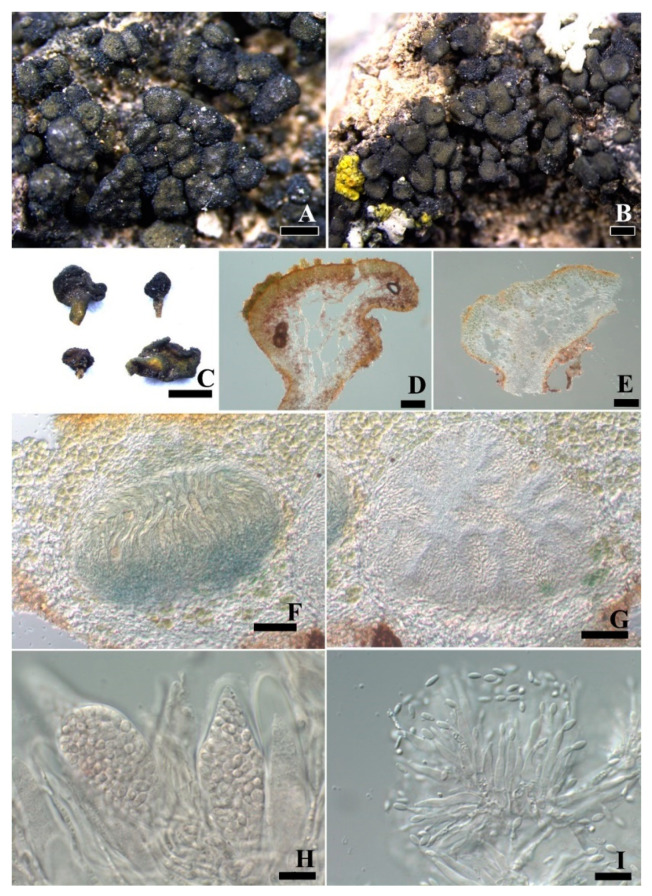
The thallus habit and the anatomic structure of *Peltula* *pseudoboletiformis* (holotype). (**A**,**B**). Squamulose thallus. (**C**). Side view of thallus with a stalk. (**D**,**E**). Thallus section with endolichenic fruiting bodies and algal layer encircled. (**F**). Immature fruiting body. (**G**). Immersed pycnidia. (**H**). Polysporous asci and elliptical ascospores. (**I**). Conidia. Bars: (**A**,**B**) = 0.5 mm, (**C**) = 1 mm, (**D**,**E**) = 100 μm, (**F**,**G**) = 50 μm, (**H**,**I**) = 10 μm.

***Peltula pseudoboletiformis*** Q.X. Yang & X.L. Wei, **sp. nov.** (Figure 6).

**MycoBank:** MB 840883.

**Etymology:** The epithet ‘*pseudoboletiformis*’ refers to the thallus morphology similar to that of the mushroom *Boletus*, which is why this new species is similar to *Peltula boletiformis*, so the epithet ‘*pseudoboletiformis*’ is chosen to define this new species.

**Typus:** CHINA, Beijing, Mentougou District, Qingshui Town, Bamuyan village, behind Xishanhong Inn, 39°51′ N, 115°33′ E, alt ca. 740 m, on rocks, 1 September 2019, X.L. Wei et al. 20,191,989 (HMAS-L145476, holotype).

**Diagnosis:** This new species is different from the similar species *P. boletiformis* in more yellow–green umbilici and larger conidia.

**Description:** Thallus saxicolous, squamulose-subfruticose, loosely or tightly clustered, up to 1.5 mm in diameter, 2 mm high, stalk 0.9 mm long in average, twisted, orange to tan; lobes circular, elliptical to angular and irregular, top almost flattened, margin entire; upper surface dark olive-green, smooth, epruinose; attached the substrate by umbilicus, isidia and soredia absent. Lobes 350–600 μm thick, upper cortex not developed, with a thin yellowish epinecral layer, 5–8 μm thick; the algal layer of immature thalli 57.5–67.3 μm thick, medulla with interwoven hyphae and small air spaces and lower cortex not clear, made up of several layers of circular cells; later algal layer encircled, 119–240 μm thick, algae in clumps of 1–4 cells, cells up to 13 × 15 μm in diameter, medulla with rare and loose hyphae and big hollow areas, hyphae 2.5–3.3 μm wide. Apothecia 1 or 2 per squamule, immersed and punctiform. Epihymenium yellow; hymenium 95–215 μm tall, paraphyses 1.5–2 μm wide, I+ wine red, K-; subhymenium up to 100 μm tall, IKI+ blue after K pretreatment; asci clavate to obclavate, 70–95 × 18–20.5 μm, with a lacerate gelatinous sheath, more than 100 spores, ascus wall IKI+ blue after K pretreatment; ascospores hyaline, ellipsoid, simple, 4.5–5.5 × 3–3.8 μm. Pycnidia immersed, cerebriform. Conidia ellipsoid, hyaline, 3.5–4.1(4.5) × 1.6–2.3 μm.

**Chemistry:** No substances detected by TLC.

**Habitat and distribution:** This species colonizes on calcareous rocks exposed to the sun, and is often in the grooves of the stone where rainwater and soil occasionally accumulate. It is known only in China up to now.

Additional specimens examined: CHINA, Beijing, Mentougou District, Qingshui Town, Bamuyan village, behind Xishanhong Inn, 39°51′ N, 115°33′ E, alt ca. 740 m, on rocks, 1 September 2019, X.L. Wei et al. 20,191,994 (HMAS-L145478), 20,192,002 (HMAS-L145477).

**Notes:** In the early stage, the thallus of this new species is squamulose attached to the substrate by the umbilicus. Gradually, the umbilicus elongated and thickened into a stalk. In the process of growing, the internal structure of thalli also changed. The boundary between the algal layer and the medulla becomes distinct, the medulla tends to be surrounded by the algal layer and the medullary cavities correspondingly become larger, varying from the previous small air spaces to the later large medullary cavities (Figure 6D,E). *Peltula pseudoboletiformis* is similar to *P. boletiformis*; the only external minor difference is the colour of thalli. *Peltula pseudoboletiformis* has a dark olive-green lobe and yellow–green umbilicus of mature thalli, while the lobe of *P. boletiformis* is black to black–brown, and the umbilicus is olive-brown. However, the genetic distance in the phylogenetic tree and the additional species delimitation analysis support them to be separate species. Therefore, the distinction between *P. pseudoboletiformis* and *P. boletiformis* mainly depends on the DNA sequences.

**Figure 7:**
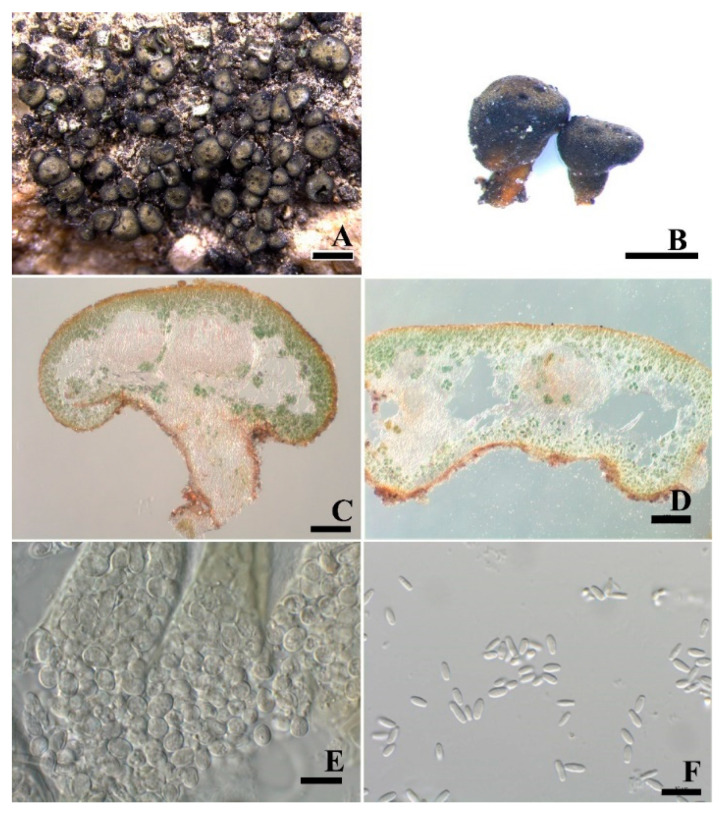
The thallus habit and the anatomic structure of *Peltula* *submarginata* (holotype). (**A**). Squamulose thallus. (**B**). Side view of thallus with a stalk. (**C**). Thallus section with endolichenic fruiting bodies and encircled algal layer. (**D**). Polysporous ascus. (**E**). Elliptical ascospores. (**F**). Conidia. Bars: (**A**) = 1 mm, (**B**) = 0.5 mm, (**C**,**D**) = 100 μm, (**E**,**F**) = 10 μm.

***Peltula submarginata*** Q.X. Yang & X.L. Wei, **sp. nov.** (Figure 7).

**MycoBank:** MB 840884.

**Etymology:** The epithet ‘*submarginata*’ refers to the thallus morphology of this new species very similar to *Peltula marginata*.

**Typus:** CHINA, Beijing, Changping District, Yanshou town, Beijing Dayangshan National Forest Park, 40°18′ N, 116°25′ E, alt 390 m, on soil of rock surface, 18 June 2019, Q.X. Yang & W.C. Wang 20,191,610 (HMAS-L145479, holotype).

**Diagnosis:** This species is similar to *P. marginata* but differs in the epinecral layer covering the whole surface and the underlying algal layer surrounding the whole perimeter of squamules, larger conidia, and absence of lower cortex.

**Description:** Thallus saxicolous, squamulose-subfruticose, clavate when young and later peltate; lobes circular to elliptical, top almost flattened, 0.3–0.8 (–2) mm in diameter, fertile lobes bigger than sterile lobes; margin entire; upper surface yellowish olive with black–brown border, smooth, epruinose; isidia and soredia absent, attached the substrate by stalk or umbilicus. Lobes 230–370 μm thick, cortex not developed but with a yellowish epinecral layer, up to 8 μm thick; algal layer encircled, 42–96 μm thick, cyanobiont unicellular, cells 8.5–10.6 μm in diameter, in clusters of 2–4 cells; medulla with rare hyphae and hollow areas in the central part, hyphae 2.2–2.4 μm thick. Apothecia 1–7 per lobe, completely immersed, discs punctiform, black. Epihymenium light yellow; hymenium up to 180 μm tall, I+ wine red, K-, paraphyses 1.7 μm wide; subhymenium up to 85 μm tall, IKI+ blue after K pretreatment; asci clavate to obclavate, with a lacerate gelatinous sheath, 112–128 × 18–20.5 μm, more than 100-spored, ascus wall IKI+ blue after K pretreatment; ascospores hyaline, ellipsoid, simple, 5.5–6.2 × 4.2–5.4 μm; subhymenium c. 85 μm thick. Pycnidia immersed, cerebriform; conidia ellipsoid to short bacilliform, hyaline, (2.1) 4.1–6.2 × 1.4–2.3 μm.

**Chemistry:** No substances detected by TLC.

**Habitat and distribution:** This species sample was found on sun-exposed sandstone, and it occupies most of the rock face where no other lichens are nearby. It is known only in China up to now.

Additional specimens examined: CHINA, Beijing, Changping District, Yanshou town, Dayangshan National Forest Park, 40°18′ N, 116°25′ E, alt 390 m, on soil of rock surface, 18 June 2019, Q.X. Yang & W.C. Wang 20,191,608 (HMAS-L145480), 20,191,612 (HMAS-L145481).

**Notes:** This new species is easily confused with *P. marginata* due to their similarity in general morphology, such as the colour of thalli, the shape of lobes and the size and number of apothecia. However, they clearly differ in presence of surrounding epinecral and algae layers and the absence of an algal layer in the lower cortex. Büdel [11] mentioned that the algal layer of *P. marginata* is obviously stratified, while the epinecral layer of *P. submarginata* covers the whole surface and the underlying algal layer surrounds the whole perimeter of squamules (Figure 7C,D). The epinecral and lower cortex layers are not distinguished but forming a whole. This feature can be also seen in *Peltula cylindrica*, but the latter species has cylindrical lobes [10]. In the phylogenetic tree, *P. submarginata* and *P. marginata* are in relatively close clades, supporting them as the most similar species. Indeed, the new species *P. submarginata* is also similar to the new species *P. pseudoboletiformis* in growth types. However, more regular squamules, brighter upper surface, black punctiform apothecia and rounded algae layer are observed in *P. submarginata* compared to irregular and dark olive-green squamules, rare apothecia, and clearly stratified or slightly surrounded algae layer in *P. pseudoboletiformis*.

## 4. Discussion

*Peltula* occurs worldwide in semiarid/arid regions and mainly grows on the fully exposed rock or soil surfaces, on seepage faces, at the border of small rivulets in the spray water region or along rain-water tracks [3,4,5,6,7,14]. Species of *Peltula* have different preferences to the growth environment. Saxicolous species of *Peltula* are more distributed in alternating dry and wet areas, while terricolous species are more distributed in desert areas with low rainfall. All the five new species in this study were collected from Beijing area, where has a typical temperate monsoon climate with obviously dry and rainy seasons and is a suitable habitat for saxicolous species, especially for those growing on calcareous rocks. In addition to the five new species, *Peltula euploca*, *P. placodizans* and *P. obscurans* etc. were also found here. Based on our current investigation and analysis, it is speculated that Beijing area may be a distribution hotspot with high species diversity of *Peltula* in China.

Subfruticose growth type has been considered as the most advanced trait in Peltulaceae [11,12,44]. The factors affecting the distribution of squamulose-subfruticose *Peltula submarginata* and *P. pseudoboletiformis* may be same to the other subfruticose species of *Peltula*, such as water conditions and light. *Peltula submarginata* and *P. pseudoboletiformis* are similar to *P. marginata* and *P. boletiformis*, respectively, among which the latter two were described to be squamulose species originally by Büdel [11] and were further defined into squamulose-semifruticose growth form by Kuaff et al. [12]. In this study, we defined these two new species as squamulose-subfruticose growth type considering the small to large medullary cavities inside the thalli [12]. They have small lobes and thick stalks, but these traits are not stable. Lobes are generally not developed when young, then the lobes enlarge, and small umbilicus gradually develop into thick and long stalks. The phylogenetic tree also shows that *P. submarginata* and *P. pseudoboletiformis* clustered in a moderately supported clade together with *P. tortuosa*, *P. lingulata* and *P. clavata* (Figure 2), which have a subfruticose growth type.

Due to different water conditions in different regions or growth environments, the subfruticose growth type may be an intermediate type between the clavate type and lingulate type or peltate type [11]. Severe ecological environments or different habitats would affect anatomical and morphological characters of lichens, which means that lichens will have a correspondingly adaptive evolution to the different environments [45,46,47,48]. Wessels and Büdel [48] summarized that black subfruticose species of *Peltula* seem to grow specifically near water sources where they can even be submerged for short periods. Combining drought and water needs, subfruticose species of *Peltula* possess both drought periods and sufficiently short water supply periods. Instantaneously sufficient water makes the thallus grow higher, while seasonal drought limits the endless growth of the thallus into fully fruticose, and the alternation between sufficient water and rapid drying promotes the generation of bubbles or cavities in the medulla to store water and exchange gas [11,47,48]. Obviously, the subfruticose species of *Peltula* can be fully drought resistant before sufficient water comes.

In addition to the specific water supply, light may also play a decisive role in the distribution of *Peltula* [11,49]. Therefore, to obtain a higher ecological niche and get more available light sources, the algae layer of subfruticose species such as *Peltula cylindrica*, *P. tortuosa* and *P. submarginata* might evolve into encircled algae layer with a larger area than the flat algae layer. These adaptive evolutionary traits in morphology and anatomy could help the species of *Peltula* colonize both arid and humid habitats well.

In China, there is still a large gap in recognizing *Peltula* and the whole Lichinales. This *Peltula* study may also be a tip of the iceberg. Therefore, it should be noted that it is necessary to add more molecular data of more species and more fresh specimens to better understand and solve the existing uncertainty within this genus. For example, the results of species delimitation analyses in this study showed that *Peltula euploca* and *P. euploca* ssp. *sorediosa*, which have always been considered to be the same species previously, are two separate ones. Although we cannot immediately deny the previous conclusions based on our results, the question of *Peltula eupcola* and *P. eupcola* ssp. *sorediosa* being conspecific or not, attracted our attention. In addition, we also found that *Peltula impressula* has great intraspecific differences in the phylogenetic tree compared with what we have understood in the past. The phylogenetic differences may be related to the colour and size of thalli. However, the specimens within the range of phylogenetic differences all have peltate and strongly rhizoid thallus, and dotted upper surface with shallow, K+ reddish violet upper cortex [19]. We consider that these differences tend to be intraspecific more than interspecific. It is believed that molecular data can allow us to better understand the intra- and inter-species differences and distances. Besides, more comprehensive and extensive research in the future will greatly improve the taxonomic status of *Peltula* and Lichinales.

## Figures and Tables

**Table 1:** Specimens used for DNA extraction and GenBank accession number of all samples used in this study. In voucher information, B.B. refers to B. Büdel, Kaiserslautern, Germany; M.S. means M. Schultz, Hamburg, Germany; K.K. is K. Kalb, Neumarkt, Germany; DUKE means Duke University.

Species	Voucher Information	GenBank Accession Numbers		
		18S	28S	ITS
*Peltula africana*	South Africa, 1990, B.B. 14304b	MF766261	MF766384	MF766343
*P. auriculata*1	Venezuela, 1992, B.B. 24901	—	DQ832330	DQ832329
*P. auriculata*2	Venezuela, 1992, B.B. 24902	MF766262	MF766385	MF766344
*P. bolanderi*	Mexico, 1993, B.B. 20196e	MF766263	MF766386	MF766345
*P. boletiformis*	South Africa, 2003, B.B. 14911a-1	MF766264	MF766387	MF766346
*P. capensis*	South Africa, 1994, B.B. 14382b 2	MF766265	MF766388	MF766347
*P. clavata*	Australia, 1987, DUKE 164 (18047a)	—	MF766389	MF766348
*P. congregata*	South Africa, 2003, B.B. 14909b-1	MF766267	MF766390	MF766349
*P. coriacea*	South Africa, 2003, B.B. 14500a-1	MF766268	MF766391	MF766350
*P. crispatula*	Morocco, 1987, B.B. 21001a	MF766269	MF766392	MF766351
*P. cylindrica*	South Africa, 2003, B.B. 14920a-1	MF766270	MF766393	MF766352
*P. euploca*	Mexico, 1993, B.B. 20162a	MF766271	MF766394	MF766353
*P. euploca* ssp. *sorediosa*	South Africa, 2003, B.B. 14921c-1	MF766272	MF766395	MF766354
*P. farinosa*	Mexico, 1993, B.B. 20119a	MF766273	MF766396	MF766355
*P. hassei*	South Africa, 1994, B.B. 14354a	MF766283	MF766406	MF766365
*P. imbricata*	Australia, 1987, B.B. 18060a	MF766274	MF766397	MF766356
*P. impressa*	Mexico, 1993, B.B. 20140f	MF766275	MF766398	MF766357
*P. inversa*	Namibia, 2001, Pretoria 15058	MF766276	MF766399	MF766358
*P. leptophylla*	Mexico, 1993, B.B. 20128a	MF766277	MF766400	MF766359
*P. lingulata*	South Africa, 1994, B.B. 14452a	MF766278	MF766401	MF766360
*P. marginata*	South Africa, 2003, B.B. 14920d-1	MF766279	MF766402	MF766361
*P. michoacanensis*	Mexico, 1993, B.B. 201401	MF766280	MF766403	MF766362
*P. obscuratula*	Morocco, 1987, B.B. ex Murcia	MF766284	MF766407	MF766366
*P. omphaliza*	Mexico, 1993, B.B. 20148b	MF766285	MF766408	MF766367
*P. patellata*	Mexico, 2003, M.S. 16254b	MF766286	MF766409	MF766368
*P. placodizans*	Mexico, 1993, B.B. 20112a	MF766287	MF766410	MF766369
*P. psammophila*	Canary Islands, 1985, BM 761074	MF766288	MF766411	MF766370
*P. radicata*	Yemen, 2002, M.S. 14241a	MF766289	MF766412	MF766371
*P. richardsii*	Mexico, 1993, B.B. 20194a	MF766290	MF766413	MF766372
*P. rodriguesii*	Namibia, 1990, B.B. 15901	MF766291	MF766414	MF766373
*P. santessonii*	South Africa, 2003, B.B. 14912b-1	MF766292	MF766415	MF766374
*P. sonorensis*	Mexico, 1993, B.B. 20196d	MF766293	MF766416	MF766375
*P. tortuosa*	Venezuela, 1996, B.B. 24039b	MF766294	MF766417	MF766376
*P. umbilicata*	South Africa, 2003, B.B. 14901a-1	DQ782887	DQ832334	DQ832333
*P. zahlbruckneri*	Mexico, 1993, B.B. 20157a	—	MF766418	MF766377
*P. corticola*	Yemen, 2002, M.S. 14201	MF766296	MF766419	MF766378
*P. steppae*	Venezuela, 1989, K.K. 23948	MF766297	MF766420	MF766379
*P. brasiliensis*	South Africa, 1983, B.B. 14083a	MF766298	MF766421	MF766380
*P. cataractae*	Dem. Rp. Congo, 1947, B.B. 1329	MF766299	—	MF766381
*Lichinella iodopulchra*	USA, 2003, M.S. 16319a (NM)	MF766300	DQ782916	MF766382
*Peltula polycarpa*	20191855 (HMAS-L 145471)	**MT499282**	**MT499319**	**MT499300**
*P. polycarpa*	20191856 (HMAS-L 145472)	**MT499286**	**MT499320**	**MT499301**
*P. polycarpa*	20191857 (HMAS-L 145473)	**MT499287**	**MT499321**	**MT499302**
*P. lobulata*	20191471 (HMAS-L 145468)	—	**MT499313**	**MT499291**
*P. lobulata*	20191474 (HMAS-L 145469)	—	**MT499314**	**MT499292**
*P. lobulata*	20191639 (HMAS-L 145470)	—	**MT499315**	**MT499293**
*P. polyphylla*	20192014 (HMAS-L 145474)	—	**MT499325**	**MT499304**
*P. polyphylla*	20191645 (HMAS-L 145475)	—	**MT499326**	**MT499303**
*P. submarginata*	20191610 (HMAS-L 145479)	**MT499284**	**MT499317**	**MT499296**
*P. submarginata*	20191608 (HMAS-L 145480)	**MT499283**	**MT499316**	**MT499294**
*P. submarginata*	20191612 (HMAS-L 145481)	**MT499285**	**MT499318**	**MT499295**
*P. pseudoboletiformis*	20191989 (HMAS-L 145476)	**MT499288**	**MT499322**	**MT499297**
*P. pseudoboletiformis*	20191994 (HMAS-L 145478)	**MT499289**	**MT499323**	**MT499298**
*P. pseudoboletiformis*	20192002 (HMAS-L 145477)	**MT499290**	**MT499324**	**MT499299**

Notes: Newly generated sequences are in bold font. “—” indicates that the corresponding sequence is absent.

**Table 2:** Key to the Species of *Peltula* in China.

1. Thallus sorediate	2
1′. Thallus esorediate	4
2. Thallus peltate; margin entire or lobed; soredia marginal	3
2′. Thallus areolate-placodiform; margin effigurate; soralia superficial, capitate	*P. placodizans*
3. Thallus small (1–2 mm in diameter), thin; upper surface dark olive-green; margins deeply lobed and undulate; squamules ascending; apothecia rare, with a raised rim when mature	*P. bolanderi*
3′. Thallus larger (to 12 mm in diameter), usually thicker; upper surface olive to olive-brown; margins entire or deeply lobed; squamules downrolled; apothecia often absent, when present, immersed and disc punctiform	*P. euploca*
4. Thallus peltate, squamulose or areolate; lobes horizontal	5
4′. Thallus dwarf subfruticose to fruticose; lobes upright	16
5. Apothecia with wide discs, usually with thalloid rims; thallus squamulose	6
5′. Apothecia immersed, usually punctiform; thallus peltate, squamulose or areolate	8
6. Squamules convex or bending down; apothecia numerous	*P. polycarpa*
6′. Squamules convex or concave; apothecia 1 (rarely 2) per squamule	7
7. Thallus polyphyllous, composed of abundant small olive-brown to olive-black lobes, up to 2.5 mm in diameter, attached to the substrate by a large and strong umbilicus cluster composed of umbilicus of each lobe	*P. polyphylla*
7′. Thallus monophyllous, rosette-lobulate, not more than 2 mm in diameter, attached to the substrate by a small umbilicus	*P. obscurans*
8. On rock	9
8′. Usually, on soil	15
9. Upper surface dark brown to black	10
9′. Thallus light or dark olive-green	11
10. Thallus squamulose, areole-like, minute, 0.3–0.5 mm wide	*P. minuta*
10′. Thallus peltate, up to 8 mm wide; squamules with a leathery gloss (and not of leathery consistency)	*P. coriacea*
11. Thallus peltate or squamulose	12
11′. Thallus squamulose-subfruticose	14
12. Hymenium K + intensely violet red or reddish violet or rose colored; lobules absent	13
12′. Hymenium K -; lobules abundant	*P. lobulata*
13. Squamules attached by a short umbilicus; hymenium with rich content of oil, K + intensely violet red or reddish violet	*P. olifera*
13′. Squamules tightly attached, nearly adnate; Hymenium without oil, K + rose colored	*P. zabolotnoji*
14. Squamules round, yellowish olive with black–brown border, in section with a ring-form algal layer; apothecia numerous and obvious, black punctiform;; umbilicus brown to black	*P. submarginata*
14′. Squamules irregularly round to elongate, dark olive-green; algae layer confined to the upper side of squamules; apothecia rare; umbilicus yellow–green	*P. pseudoboletiformis*
15. Thallus shield-like; upper surface K + violet purple	*P. radicata*
15′. Thallus squamulose, partly imbricate with numerous minute impressions on the surface; upper cortex K + pinkish-violet	*P. impressula*
16. Thallus olive black to black, usually isidiate; lobes rarely branched	*P. clavata*
16′. Thallus olive green to black, isidia absent; lobes usually branched	17
17. Thallus little branched	18
17′. Thallus richly branched	*P. tortuosa*
18. Thallus olive-brown with lighter-colored tips, 0.5–4 mm high; apothecia terminal, immersed	*P. cylindrica*
18′. Thallus olive to black with lighter-colored base, 5–8 mm high; apothecia not seen	*P. applanata*

## Data Availability

Publicly available datasets were analyzed in this study. The multilocus data alignment file was deposited in TreeBASE (http://www.treebase.org, accessed on 17 December 2021; accession number 26385). All newly generated sequences were deposited in GenBank (https://www.ncbi.nlm.nih.gov/genbank/, accessed on 17 December 2021; Table 1). All new taxa were deposited in MycoBank (https://www.mycobank.org/, accessed on 17 December 2021).

## Supplementary Materials

The following supporting information can be downloaded at: www.mdpi.com/article/10.3390/jof8020134/s1, Figure S1: The Bayesian tree of *Peltula* species based on the concatenated ITS + nuSSU + nuLSU (three-gene) data set; Figure S2: The maximum likelihood tree of *Peltula* species based on the nuSSU sequences; Figure S3: The maximum likelihood tree of *Peltula* species based on the ITS sequences; Figure S4: The maximum likelihood tree of *Peltula* species based on the nuLSU sequences; Table S1: ABGD species delimitation results; Table S2: bPTP species delimitation results based on Maximum Likelihood partition; Table S3: GMYC species delimitation results.
