# Supplementary material for: Five New Species of the Lichen-Forming Fungal Genus Peltula from China
Source: J Fungi (Basel). 2022 Jan 28;8(2):134. doi: 10.3390/jof8020134 (PMC8878757; doi:10.3390/jof8020134)
Supplement: Supplementary file 1 [file jof-08-00134-s001.zip › jof-1538512-supplementary.pdf]

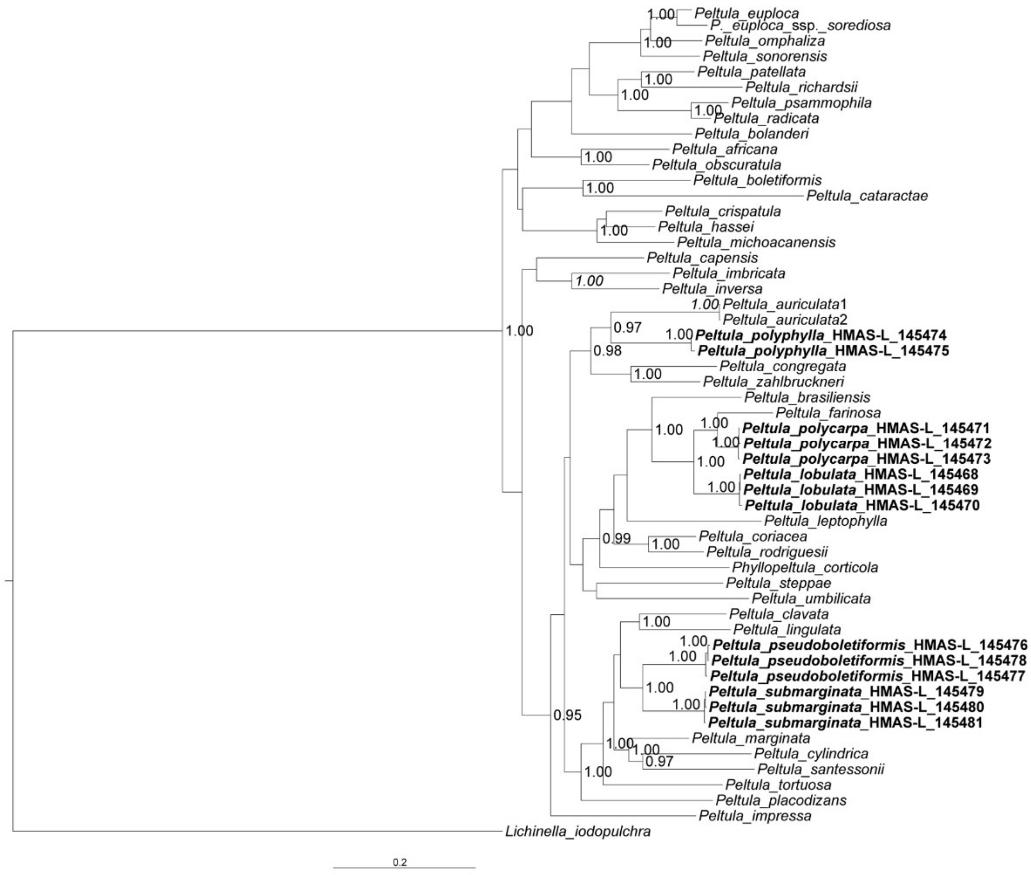

Figure S1. The Bayesian tree of *Peltula* species based on the concatenated ITS + nuSSU + nuLSU (three-gene) data set. The numbers in each node represent posterior probability (PP) values. Posterior probability values ≥ 0.95 were plotted on the branches of the MrBayes tree. The clades corresponding to the new species are in bold, which indicate that these sequences are newly generated for this study. Scale = 0.2 substitution per site.

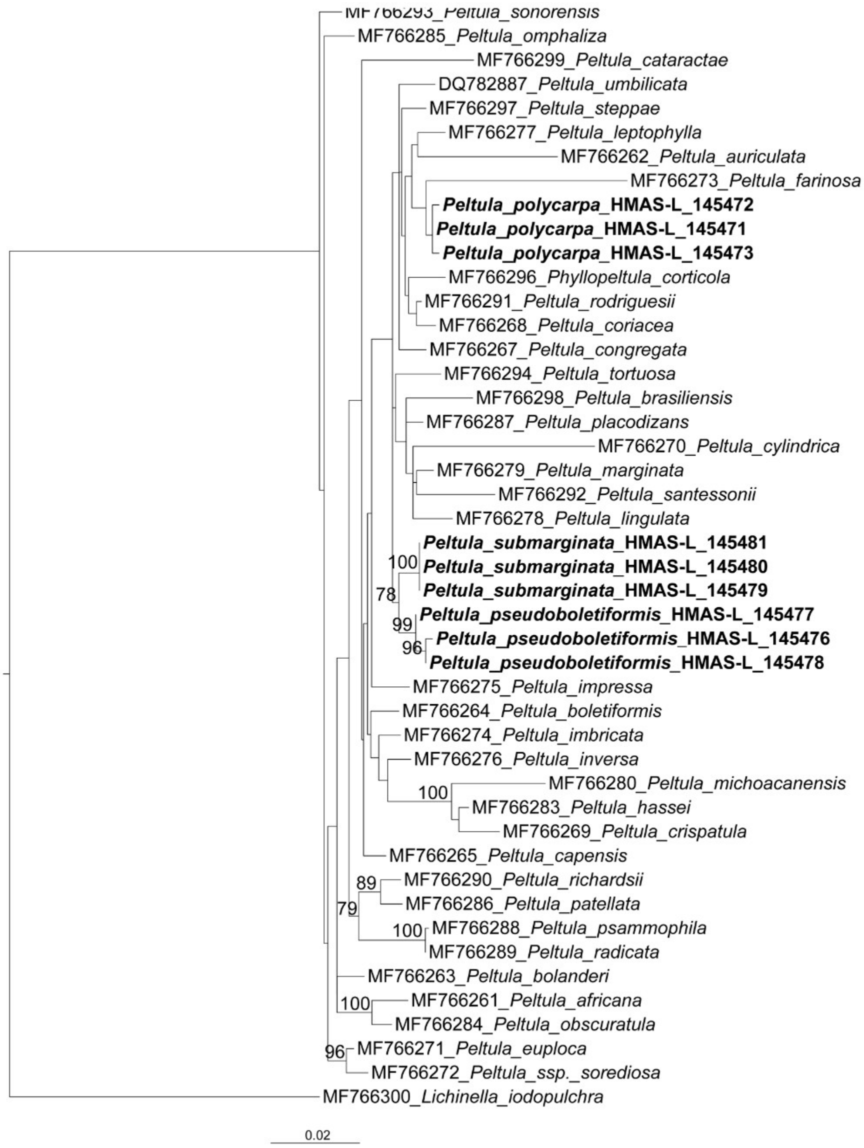

Figure S2. The maximum likelihood tree of *Peltula* species based on the nuSSU sequences. The numbers in each node represent bootstrap support (BS). Bootstrap values ≥ 75 were plotted on the branches of the RAxML tree. The clades corresponding to the new species are in bold, which indicate that these sequences are newly generated for this study. Scale = 0.02 substitution per site.

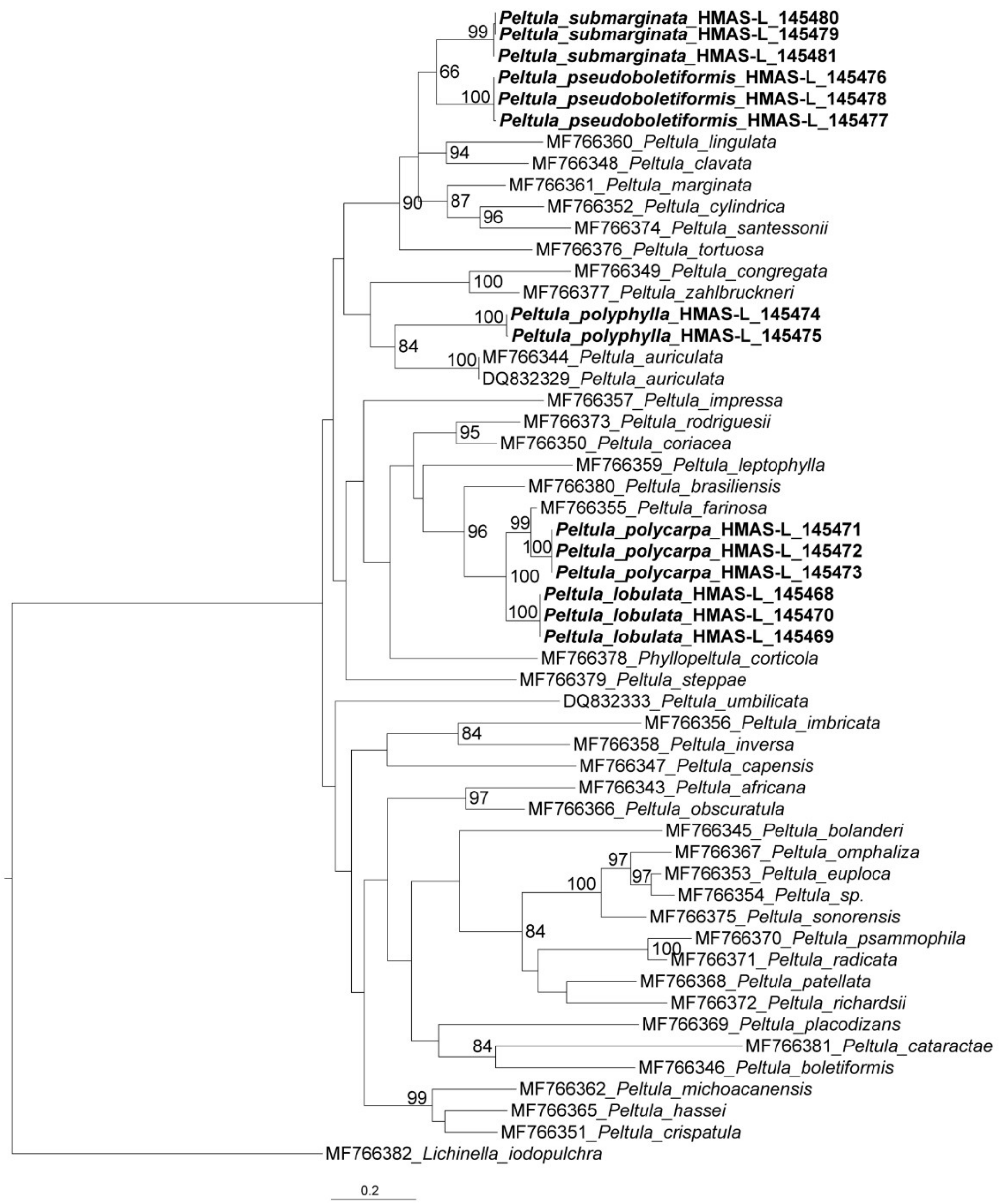

Figure S3. The maximum likelihood tree of *Peltula* species based on the ITS sequences. The numbers in each node represent bootstrap support (BS). Bootstrap values ≥ 75 were plotted on the branches of the RAxML tree. The clades corresponding to the new species are in bold, which indicate that these sequences are newly generated for this study. Scale = 0.2 substitution per site.

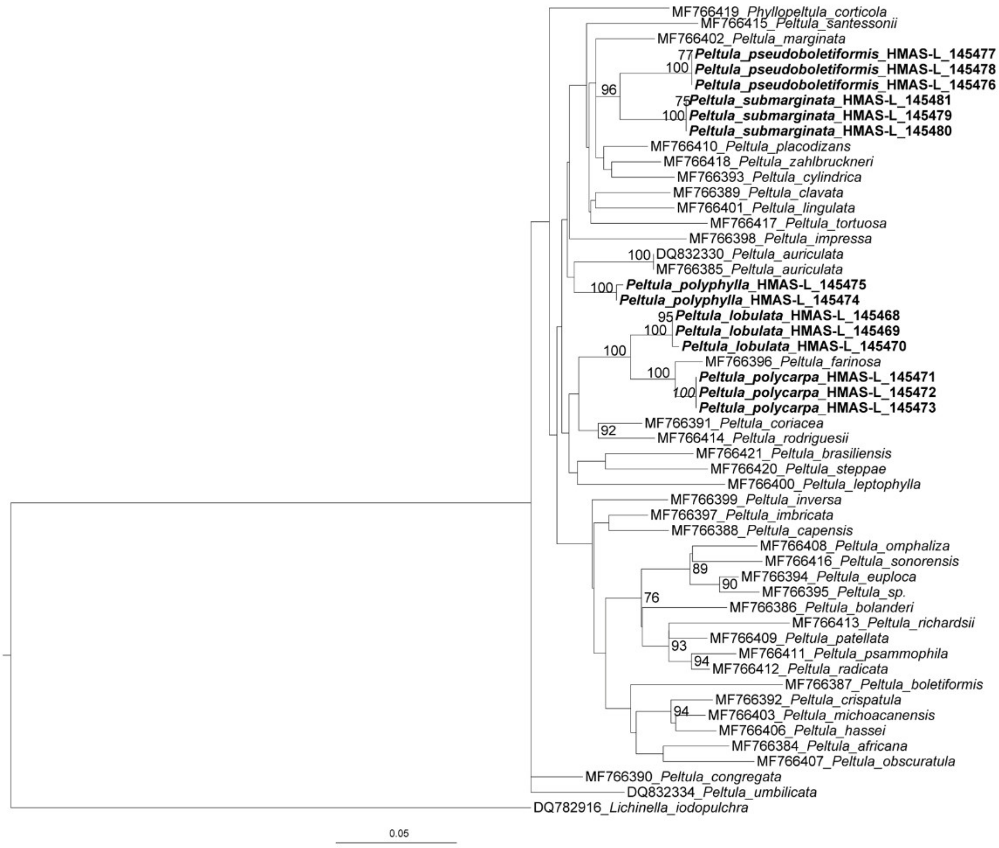

Figure S4. The maximum likelihood tree of *Peltula* species based on the nuLSU sequences. The numbers in each node represent bootstrap support (BS). Bootstrap values ≥ 75 were plotted on the branches of the RAxML tree. The clades corresponding to the new species are in bold, which indicate that these sequences are newly generated for this study. Scale = 0.05 substitution per site.

Table S1. ABGD species delimitation results

Parameter settings	P0.001-0.1, X=0.5	P0.001-0.01, X=1
Delimited species	**Group[1] n: 1 ;**id: Lichinella_iodopulchra	**Group[1] n: 1 ;**id: Lichinella_iodopulchra
	**Group[2] n: 1 ;**id: P._euploca_ssp._sorediosa	**Group[2] n: 1 ;**id: P._euploca_ssp._sorediosa
	**Group[3] n: 1 ;**id: Peltula_africana	**Group[3] n: 1 ;**id: Peltula_africana
	**Group[4] n: 2 ;**id: Peltula_auriculata1 Peltula_auriculata2	**Group[4] n: 2 ;**id: Peltula_auriculata1 Peltula_auriculata2
	**Group[5] n: 1 ;**id: Peltula_bolanderi	**Group[5] n: 1 ;**id: Peltula_bolanderi
	**Group[6] n: 1 ;**id: Peltula_boletiformis	**Group[6] n: 1 ;**id: Peltula_boletiformis
	**Group[7] n: 1 ;**id: Peltula_brasiliensis	**Group[7] n: 1 ;**id: Peltula_brasiliensis
	**Group[8] n: 1 ;**id: Peltula_capensis	**Group[8] n: 1 ;**id: Peltula_capensis
	**Group[9] n: 1 ;**id: Peltula_cataractae	**Group[9] n: 1 ;**id: Peltula_cataractae
	**Group[10] n: 1 ;**id: Peltula_clavata	**Group[10] n: 1 ;**id: Peltula_clavata
	**Group[11] n: 1 ;**id: Peltula_congregata	**Group[11] n: 1 ;**id: Peltula_congregata
	**Group[12] n: 1 ;**id: Peltula_coriacea	**Group[12] n: 1 ;**id: Peltula_coriacea
	**Group[13] n: 1 ;**id: Peltula_crispatula	**Group[13] n: 1 ;**id: Peltula_crispatula
	**Group[14] n: 1 ;**id: Peltula_cylindrica	**Group[14] n: 1 ;**id: Peltula_cylindrica
	**Group[15] n: 1 ;**id: Peltula_euploca	**Group[15] n: 1 ;**id: Peltula_euploca
	**Group[16] n: 1 ;**id: Peltula_farinosa	**Group[16] n: 1 ;**id: Peltula_farinosa
	**Group[17] n: 1 ;**id: Peltula_hassei	**Group[17] n: 1 ;**id: Peltula_hassei
	**Group[18] n: 1 ;**id: Peltula_imbricata	**Group[18] n: 1 ;**id: Peltula_imbricata
	**Group[19] n: 1 ;**id: Peltula_impressa	**Group[19] n: 1 ;**id: Peltula_impressa
	**Group[20] n: 1 ;**id: Peltula_inversa	**Group[20] n: 1 ;**id: Peltula_inversa

**Group[21] n: 1 ;**id: Peltula_leptophylla	**Group[21] n: 1 ;**id: Peltula_leptophylla
**Group[22] n: 1 ;**id: Peltula_lingulata	**Group[22] n: 1 ;**id: Peltula_lingulata
**Group[23] n: 3 ;**id: Peltula_lobulata_145468 Peltula_lobulata_145469 Peltula_lobulata_145470	**Group[23] n: 3 ;**id: Peltula_lobulata_145468 Peltula_lobulata_145469 Peltula_lobulata_145470
**Group[24] n: 1 ;**id: Peltula_marginata	**Group[24] n: 1 ;**id: Peltula_marginata
**Group[25] n: 1 ;**id: Peltula_michoacanensis	**Group[25] n: 1 ;**id: Peltula_michoacanensis
**Group[26] n: 1 ;**id: Peltula_obscuratula	**Group[26] n: 1 ;**id: Peltula_obscuratula
**Group[27] n: 1 ;**id: Peltula_omphaliza	**Group[27] n: 1 ;**id: Peltula_omphaliza
**Group[28] n: 1 ;**id: Peltula_patellata	**Group[28] n: 1 ;**id: Peltula_patellata
**Group[29] n: 1 ;**id: Peltula_placodizans	**Group[29] n: 1 ;**id: Peltula_placodizans
**Group[30] n: 3 ;**id: Peltula_polycarpa_145471 Peltula_polycarpa_145472 Peltula_polycarpa_145473	**Group[30] n: 3 ;**id: Peltula_polycarpa_145471 Peltula_polycarpa_145472 Peltula_polycarpa_145473
**Group[31] n: 2 ;**id: Peltula_polyphylla_145474 Peltula_polyphylla_145475	**Group[31] n: 2 ;**id: Peltula_polyphylla_145474 Peltula_polyphylla_145475
**Group[32] n: 1 ;**id: Peltula_psammophila	**Group[32] n: 1 ;**id: Peltula_psammophila
**Group[33] n: 3 ;**id: P._pseudoboletiformis_145476 P._pseudoboletiformis_145477 P._pseudoboletiformis_145478	**Group[33] n: 3 ;**id: P._pseudoboletiformis_145476 P._pseudoboletiformis_145477 P._pseudoboletiformis_145478
**Group[34] n: 1 ;**id: Peltula_radicata	**Group[34] n: 1 ;**id: Peltula_radicata
**Group[35] n: 1 ;**id: Peltula_richardsii	**Group[35] n: 1 ;**id: Peltula_richardsii
**Group[36] n: 1 ;**id: Peltula_rodriguesii	**Group[36] n: 1 ;**id: Peltula_rodriguesii
**Group[37] n: 1 ;**id: Peltula_santessonii	**Group[37] n: 1 ;**id: Peltula_santessonii
**Group[38] n: 1 ;**id: Peltula_sonorensis	**Group[38] n: 1 ;**id: Peltula_sonorensis

	**Group[39] n: 1 ;**id: Peltula_steppae	**Group[39] n: 1 ;**id: Peltula_steppae
	**Group[40] n: 3 ;**id: P._submarginata_145479 P._submarginata_145480 P._submarginata_145481	**Group[40] n: 3 ;**id: P._submarginata_145479 P._submarginata_145480 P._submarginata_145481
	**Group[41] n: 1 ;**id: Peltula_tortuosa	**Group[41] n: 1 ;**id: Peltula_tortuosa
	**Group[42] n: 1 ;**id: Peltula_umbilicata	**Group[42] n: 1 ;**id: Peltula_umbilicata
	**Group[43] n: 1 ;**id: Peltula_zahlbruckneri	**Group[43] n: 1 ;**id: Peltula_zahlbruckneri
	**Group[44] n: 1 ;**id: Phyllopeltula_corticola	**Group[44] n: 1 ;**id: Phyllopeltula_corticola

Table S2. bPTP species delimitation results based on Maximum Likelihood partition

Species No.	Sample name	Support
**1**	Peltula_cataractae	1.000
**2**	Peltula_steppae	1.000
**3**	Peltula_umbilicata	1.000
**4**	Peltula_impressa	1.000
**5**	Peltula_boletiformis	1.000
**6**	Peltula_leptophylla	1.000
**7**	Phyllopeltula_corticola	1.000
**8**	Peltula_placodizans	1.000
**9**	Peltula_bolanderi	1.000
**10**	Peltula_tortuosa	1.000
**11**	Peltula_cylindrica	1.000
**12**	Peltula_santessonii	1.000
**13**	Peltula_marginata	1.000
**14**	Peltula_capensis	1.000
**15**	Peltula_polyphylla_145475, Peltula_polyphylla_145474	0.824
**16**	Peltula_auriculata2, Peltula_auriculata1	0.492
**17**	Peltula_patellata	1.000
**18**	Peltula_richardsii	1.000
**19**	Peltula_inversa	1.000
**20**	Peltula_imbricata	1.000
**21**	Peltula_clavata	1.000
**22**	Peltula_lingulata	1.000

**23**	Peltula_brasiliensis	1.000
**24**	Peltula_obscuratula	1.000
**25**	Peltula_africana	1.000
**26**	Peltula_zahlbruckneri	1.000
**27**	Peltula_congregata	1.000
**28**	Peltula_michoacanensis	1.000
**29**	Peltula_pseudoboletiformis_145476, Peltula_pseudoboletiformis_145478, Peltula_pseudoboletiformis_145477	0.991
**30**	Peltula_submarginata_145480, Peltula_submarginata_145479, Peltula_submarginata_145481	0.882
**31**	Peltula_sonorensis	1.000
**32**	Peltula_hassei	1.000
**33**	Peltula_crispatula	1.000
**34**	Peltula_rodriguesii	1.000
**35**	Peltula_coriacea	1.000
**36**	Peltula_polycarpa_145471, Peltula_polycarpa_145472, Peltula_polycarpa_145473	0.932
**37**	Peltula_farinosa	0.999
**38**	Peltula_lobulata_145468, Peltula_lobulata_145469, Peltula_lobulata_145470	0.850
**39**	Peltula_omphaliza	1.000
**40**	Peltula_radicata	0.999
**41**	Peltula_psammophila	0.999
**42**	Peltula_euploca_ssp._sorediosa	0.996
**43**	Peltula_euploca	0.996

Table S3. GMYC species delimitation results

	Method	single
**Results summary**	Likelihood of null model	235.6642
	Maximum likelihood of GMYC model	247.529
	Likelihood ratio	23.72973
	Result of LR test	7.033232e-06***
**No.**	**Delimited species No.**	**Sample name**
**1**	1	Peltula_auriculata1
**2**	1	Peltula_auriculata2
**3**	2	Peltula_polyphylla_HMAS_L_145474
**4**	2	Peltula_polyphylla_HMAS_L_145475
**5**	3	Peltula_polycarpa_HMAS_L_145473
**6**	3	Peltula_polycarpa_HMAS_L_145471
**7**	3	Peltula_polycarpa_HMAS_L_145472
**8**	4	Peltula_lobulata_HMAS_L_145470
**9**	4	Peltula_lobulata_HMAS_L_145468
**10**	4	Peltula_lobulata_HMAS_L_145469
**11**	5	Peltula_pseudoboletiformis_HMAS_L_145477
**12**	5	Peltula_pseudoboletiformis_HMAS_L_145476
**13**	5	Peltula_pseudoboletiformis_HMAS_L_145478
**14**	6	Peltula_submarginata_HMAS_L_145481
**15**	6	Peltula_submarginata_HMAS_L_145479
**16**	6	Peltula_submarginata_HMAS_L_145480
**17**	7	Lichinella_iodopulchra
**18**	8	Peltula_euploca_ssp._sorediosa
**19**	9	Peltula_euploca
**20**	10	Peltula_omphaliza
**21**	11	Peltula_sonorensis
**22**	12	Peltula_patellata
**23**	13	Peltula_richardsii
**24**	14	Peltula_psammophila
**25**	15	Peltula_radicata
**26**	16	Peltula_bolanderi
**27**	17	Peltula_africana
**28**	18	Peltula_obscuratula
**29**	19	Peltula_crispatula

**30**	20	Peltula_hassei
**31**	21	Peltula_michoacanensis
**32**	22	Peltula_boletiformis
**33**	23	Peltula_cataractae
**34**	24	Peltula_congregata
**35**	25	Peltula_zahlbruckneri
**36**	26	Peltula_umbilicata
**37**	27	Peltula_brasiliensis
**38**	28	Peltula_farinosa
**39**	29	Peltula_coriacea
**40**	30	Peltula_rodriguesii
**41**	31	Phyllopeltula_corticola
**42**	32	Peltula_leptophylla
**43**	33	Peltula_steppae
**44**	34	Peltula_clavata
**45**	35	Peltula_lingulata
**46**	36	Peltula_cylindrica
**47**	37	Peltula_santessonii
**48**	38	Peltula_marginata
**49**	39	Peltula_tortuosa
**50**	40	Peltula_placodizans
**51**	41	Peltula_impressa
**52**	42	Peltula_capensis
**53**	43	Peltula_imbricata
**54**	44	Peltula_inversa
